# Draft Genome Sequence of Megaplasmid-Bearing Staphylococcus sciuri Strain B9-58B, Isolated from Retail Pork

**DOI:** 10.1128/MRA.01474-19

**Published:** 2020-01-02

**Authors:** Leena Neyaz, Anand B. Karki, Mohamed K. Fakhr

**Affiliations:** a Department of Biological Science, The University of Tulsa, Tulsa, Oklahoma, USA; University of Rochester School of Medicine and Dentistry

## Abstract

Here, we report the genome sequence of the megaplasmid-bearing Staphylococcus sciuri strain B9-58B, isolated from retail pork. This strain contains a 2,761,440-bp chromosome and a 162,858-bp megaplasmid. The genome contains putative genes involved in virulence, the stress response, and antimicrobial agent and heavy metal resistance.

## ANNOUNCEMENT

*Staphylococcus* spp. are major foodborne pathogens that cause food poisoning through the production of enterotoxins (1). Numerous studies in the United States have revealed a high prevalence of multidrug-resistant *Staphylococcus* strains in food, indicating the potential acquisition and colonization of strains by food industry workers and consumers (2–4). We previously reported a high prevalence of Staphylococcus aureus in retail pork products, and isolates exhibited multidrug resistance and encoded toxins (5).

Staphylococcus sciuri is a coagulase-negative, oxidase-positive, and novobiocin-resistant species. While animals are the main reservoir for S. sciuri (6), this organism has also been isolated from humans, food, and the environment (4, 7, 8). Although coagulase-negative species are generally known as harmless members of the microbiota of human skin and mucous membranes, they are increasingly being identified as causative agents of health care- and community-associated infections. Recent research has proposed that S. sciuri functions as a reservoir of virulence and antibiotic resistance genes that facilitate S. aureus colonization and survival in different environments (9). Existing reports on foodborne *Staphylococcus* spp. have focused primarily on coagulase-positive S. aureus strains.

Here, we announce the draft genome sequence of S. sciuri strain B9-58B, which was previously isolated from retail pork and was initially identified as S. aureus by PCR using the S. aureus-specific primers Sa442-1 and Sa442-2 (5, 10). This isolate contains a 162,858-bp megaplasmid, which was confirmed by S1 nuclease pulsed-field gel electrophoresis, as described previously (11). This isolate was grown for 16 to 24 h in tryptic soy agar at 37°C under aerobic conditions, and genomic DNA was isolated and purified using a DNeasy blood and tissue kit (Qiagen, Valencia, CA). The library used for sequencing was prepared using the Nextera XT library preparation kit for small genomes (Illumina, Inc., San Diego, CA), and a MiSeq desktop sequencer was used to sequence the whole genome with an Illumina v2 reagent kit (2 × 250 cycles). The 500 cycles yielded approximately 229× coverage. The sequence reads that passed the initial MiSeq quality standards were assessed using CLC Genomics Workbench v12.0 (Qiagen) and were trimmed for adapters, low-quality reads, and short reads. A total of 3,202,194 paired-end reads, with a mean sequence length of 201.1 bp, were submitted to the comprehensive genome analysis service at PATRIC v3.5.39 (12) for *de novo* assembly using the default settings. The resulting contigs, with an *N*_50_ value of 249,127 bp and a GC content of 32.45%, were scaffolded by aligning them against the closest reference using Genome Finishing Module v1.9 of CLC Genomics Workbench v12.0 (Qiagen).

The genome of S. sciuri B9-58B contained a single chromosome and a megaplasmid, which were 2,761,440 and 162,858 bp, respectively, with GC contents of 32.66% and 31.13%. While it was possible to close the chromosome, the plasmid remained unclosed, with 39 contigs. The B9-58B genome was annotated using the RAST tool kit. The chromosome contained 2,712 protein-coding sequences, 62 tRNA genes, and 16 rRNA genes, while the megaplasmid had 218 coding sequences. Multiple stress response genes, including osmotic, oxidative, heat/cold shock, and periplasmic stress-related genes, in addition to detoxification and carbon starvation genes, were chromosomally located. This strain contained genes conferring resistance to a variety of antibiotics and heavy metals, such as bacitracin, fluoroquinolones, copper, cobalt-zinc-cadmium, and mercury. Interestingly, the chromosome contained a *Mycobacterium* virulence operon that is involved in protein synthesis and facilitates invasion and intracellular resistance. The megaplasmid contained genes conferring resistance to cobalt, zinc, copper, cadmium, fosfomycin, and chromium.

### Data availability.

The raw sequence reads have been deposited in the Sequence Read Archive (SRA) under accession number PRJNA555628. The whole-genome shotgun sequences for Staphylococcus sciuri strain B9-58B have been deposited in GenBank under accession number CP041879 for the chromosome and accession numbers CP041880, CP041881, CP041882, CP041883, CP041884, CP041885, CP041886, CP041887, CP041888, CP041889, CP041890, CP041891, CP041892, CP041893, CP041894, CP041895, CP041896, CP041897, CP041898, CP041899, CP041900, CP041901, CP041902, CP041903, CP041904, CP041905, CP041906, CP041907, CP041908, CP041909, CP041910, CP041911, CP041912, CP041913, CP041914, CP041915, CP041916, CP041917, and CP041918 for the plasmid.
